# Comparable results using 2.0-mm vs. 3.5-mm screw augmentation in midshaft clavicle fractures: a 10-year experience

**DOI:** 10.1186/s40001-021-00487-w

**Published:** 2021-02-02

**Authors:** M. Wurm, M. Zyskowski, F. Greve, A. Gersing, P. Biberthaler, C. Kirchhoff

**Affiliations:** 1 Department of Trauma Surgery, Klinikum Rechts Der Isar, Technical University of Munich, Ismaninger Strasse 22, 81675 Munich, Germany; 2 Department of Radiology, Klinikum Rechts Der Isar, Technical University of Munich, Munich, Germany

**Keywords:** Clavicle, Fracture, Plate, Screw, Osteosynthesis, Cortical, Alignment

## Abstract

**Purpose:**

Absence of cortical alignment in wedge-shaped and multifragmentary fractures (Fx) results in decreased fixation stability. The aim of this study was to compare the outcome using 2.0- vs. 3.5-mm screws for open reduction and internal fixation (ORIF) in dislocated, wedge-shaped or fragmentary midshaft clavicle fractures.

**Materials and methods:**

Patients suffering from AO/OTA 15 2.A-C midshaft clavicle fractures were operatively treated between 2008 and 2018. 2.0- or 3.5-mm cortical screws were used to restore anatomic alignment in dislocated, wedge-shaped and fragmentary clavicle fractures. Data of radiologic outcome were collected until fracture consolidation was identified.

**Results:**

80 consecutive patients with a mean age of 44.5 ± 16.3 years, who were operatively treated for dislocated midshaft clavicle fractures were enrolled. 40 patients were treated using 2.0-mm and 40 patients using routine 3.5-mm cortical screws, respectively. Time to fracture consolidation was 12.8 ± 7.8 months. No mal- or non-unions occurred during routine follow-up until 18 months postoperatively.

**Conclusion:**

Restoring anatomic alignment in wedge or fragmentary clavicle fractures can ultimately be addressed using cortical screw augmentation. Both groups showed comparable results with respect to fracture reduction, fixation and stability as well as time to consolidation of the fracture, while the 2.0-mm screw diameter was associated with easier handling of small Fx fragments.

## Introduction

In daily clinical routine, the majority of clavicle fractures can be treated conservatively. However, the degree of dislocation and shortening of the clavicle needs to be considered regarding decision-making. Open Fx, infection or non-union of the Fx are regarded as evident indications for surgical treatment. However, there has been a considerable increase in operative treatment of clavicle Fx even exceeding the rising incidence of clavicle Fx [1]. From a biomechanical point of view, plate osteosynthesis is superior to intramedullary nailing of the clavicle with regard to rotational stability [2]. Furthermore, superior plating revealed a more stable situation in transverse fractures compared to antero-inferior plating [2]. Wedge-shaped or fragmentary Fx are sometimes difficult to reduce. However, fragment fixation and stability are essential factors for consolidation and repair of the original anatomical state, since the lack of cortical alignment leads to a lower stability ultimately resulting in possible mal- or non-union as shown by Hulsmans et al. in 2018 [2]. Anatomic alignment can be attained through temporary fixation by using Kirschner wires (K-wires) or use of cortical screws, although stabilization should ultimately only be obtained by screws, since K-wire fixation has led to sometimes fatal complications such as migration. Current literature as well as guidelines of the “Arbeitsgemeinschaft für Osteosynthesefragen” (AO/OTA) recommend a screw diameter of 3.5 mm for these lag screws. However, in relation to the osseous geometry of the clavicle, smaller screw diameters might be sufficient. The primary hypothesis of this study was to identify if there is a difference utilizing 2.0-mm screws vs. 3.5-mm screws with respect to time until bony union. The second hypothesis was that 2.0-mm screws are superior to 3.5-mm screws when addressing smaller fracture fragments in clavicle fractures. A further aim of the presented study was to compare results of these two different cortical screw diameters (2.0 vs “standard” 3.5 mm) to restore cortical alignment in dislocated wedge-shaped or fragmentary midshaft clavicle Fx focusing on anatomic alignment and time until Fx healing.

## Materials and methods

In this retrospective cohort study, patients suffering from midshaft clavicle Fx (AO/OTA 15 2.A-C; spiral/transverse, wedge-shaped and fragmentary midshaft clavicle Fx), who were operatively treated in our level I trauma center from September 1st 2008 until January 1st 2018 were identified. The AO/OTA classification was used to determine indication for surgical treatment. Preoperative X-ray imaging in two planes was performed in all patients. Whole-body computed tomography (CT) was performed in patients who were transferred to our emergency room to rule out additional injury [3]. Operative treatment was indicated according to the degree of dislocation (> 100%) or shortening of the clavicle of more than 14 mm in females and of 16 mm in males, respectively [4]. Patients older than 18 years suffering from dislocated midshaft clavicle Fx, who completed all routine follow-up appointments (6, 12, 26 and 52 weeks postoperatively) until Fx healing were enrolled in this study. Correspondingly, patients < 18 years of age with prior operative treatment of the clavicle, dislocation less than 100% or shortening of less than 14/16 mm, polytraumatized patients or patients who did not complete all follow-up visits until fracture healing were excluded. 40 patients were treated using 2.0 mm (Aptus, Medartis, Basel, Switzerland), the other 40 patients were treated with 3.5 mm (DePuySynthes, PA, USA) cortical lag screws, respectively. Final fixation was achieved using an anatomically preformed locking compression plate (DePuySynthes, PA, USA or Arthrex, Naples, FL, USA, respectively). Radiologic and functional outcome was routinely assessed 6, 12, 26 and 52 weeks after surgery or until Fx healing was achieved after open reduction and internal fixation. Further follow-up visits were performed in patients who requested implant removal [5]. Time to healing was recorded and interpreted by two experienced orthopedic trauma surgeons (P.B, C.K.) as well as by one independent experienced radiologist (A.G.). Statistical comparison of time until fracture healing was performed for both screw types. Postoperative complications, time until fracture healing and time until implant removal was reported. Statistical workup was performed using SPSS 25 for Mac (Chicago, IL, USA). Institutional review board approval was obtained prior to this study (No.: 2/20S, Ethical Committee of the Technical University of Munich).

## Results

**Fig. 1:**
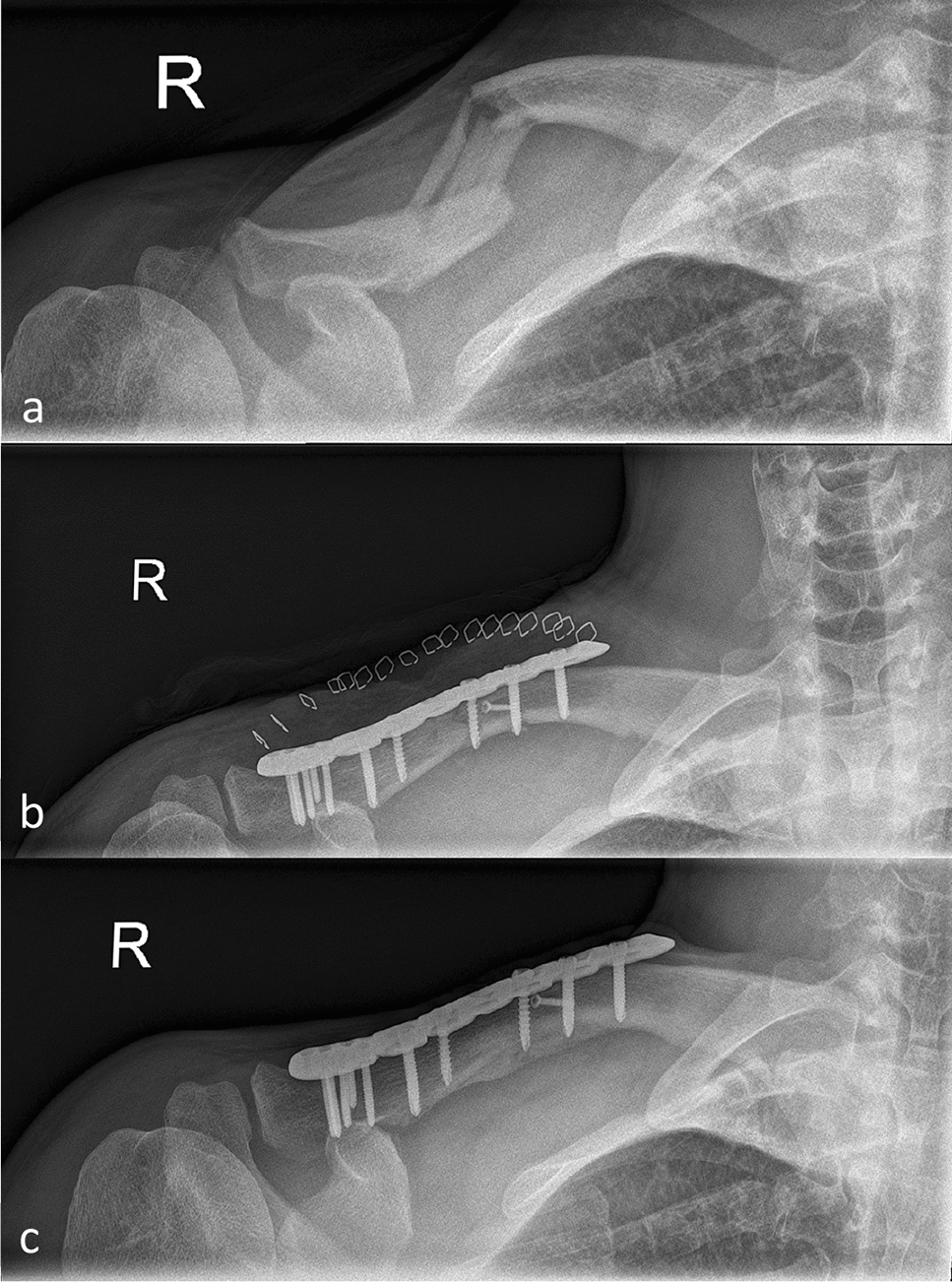
X-ray images of a fragmentary (OTA 2.C) midshaft clavicle fracture treated with 2.0-mm cortical screw augmentation and locking compression plate. Preoperative (**a**), postoperative (**b**) and images after fracture consolidation

80 patients (63 male, 17 female) with a mean age of 44.5 ± 16.3 (range 25.1–81.01) years were operatively treated for dislocated midshaft clavicle Fx (AO/OTA 15.2A-C) between September 1st 2008 and January 1st 2018. The patients showed a comparable mean age of 43.6 ± 16.0 (male) compared to 45.8 ± 15.4 (female) (*p* = 0.969). 42 Fx occurred on the left and 38 on the right hand side. 40 consecutive patients were assigned to the 2.0-mm cortical screw group (group I; men/women *n* = 32/8) and another 40 consecutive patients were treated by 3.5 mm (group II; men/women *n* = 31/9) cortical lag screw augmentation. Overall, 80 consecutive patients were enrolled for statistical follow-up. 25 of 40 (62.5%) group I patients and 32 of 40 (80%) group II patients completed all follow-up visits and were eventually considered for statistical evaluation (Table 1 and Fig. 1).

**Table 1:** Demographics, descriptive statistics and AO/OTA classification of enrolled patients with dislocated midshaft clavicle fractures

Operative treatment of dislocated midshaft clavicle fractures (n = 80)					
	Men		Women	Time until Fx consolidation (overall)	
Group I (n = 40)	32		8	10.5 ± 5.5	*p* = 0.10 (n.s.)
Group II (n = 40)	31		9	14.6 ± 8.5	
	Men	Overall	Women		
Age (overall)	43.63 ± 16.0	44.5 ± 16.3	45.8 ± 15.4		*p* = 0.969(n.s)
Fracture classification (AO/OTA)	15.2A		15.2B	15.2C	
	n = 7		n = 52	n = 21	
Mean operation duration (min)	Group I		Overall	Group II	
	83.1 ± 19.2		88.7 ± 29.1	93.4 ± 35.8	*p* = 0.013 (s.)
Implant removals (n =)	8/40 (20%)		33/80 (41,25%)	25/40 (62,5%)	
Time to implant removal (months)	21.4 ± 5.4		18.9 ± 7.1	17.7 ± 7.4	*p* = 0.705 (n.s)
	Left		Right		
Fracture site	38		42

**Fig. 2:**
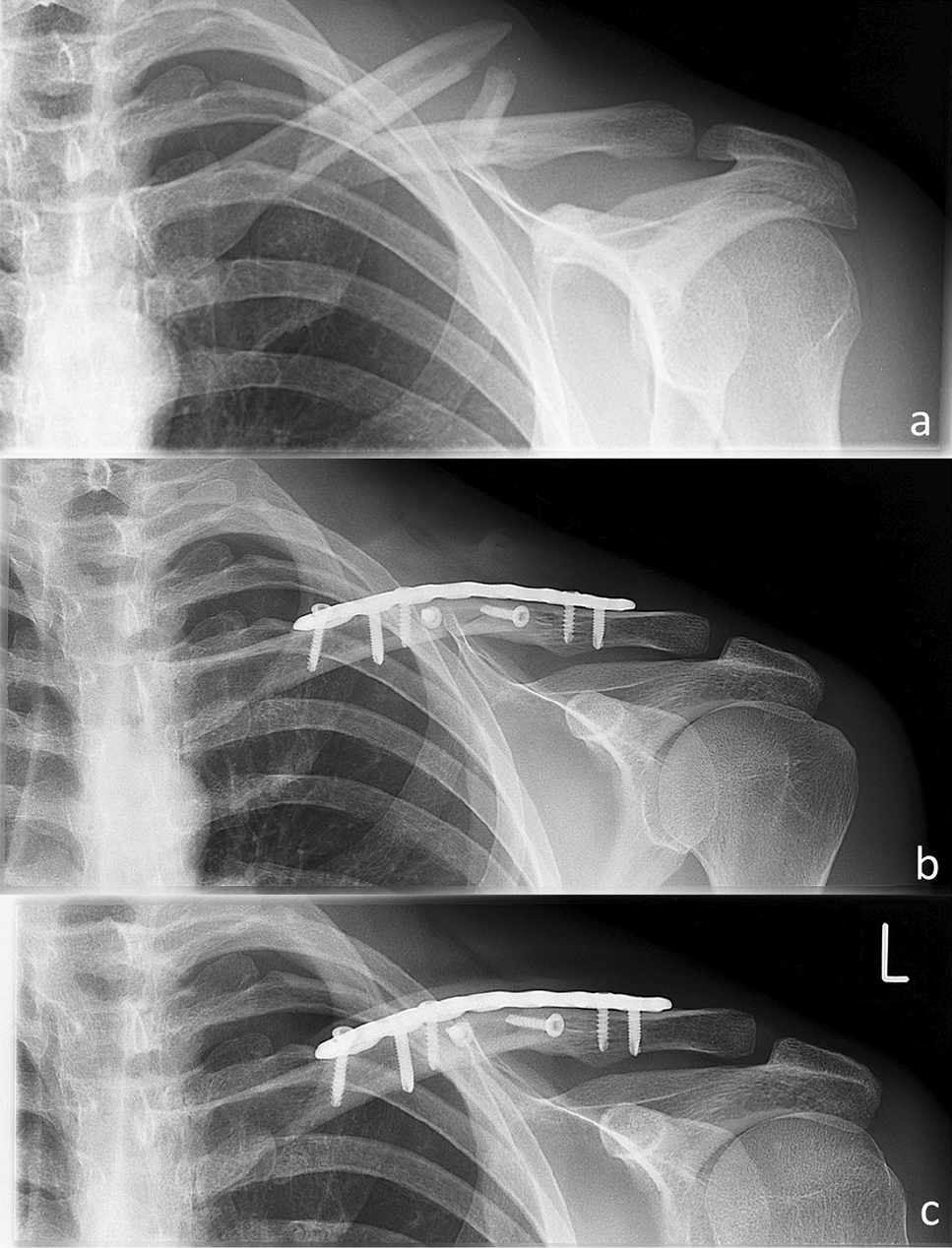
Preoperative (**a**) and postoperative (**b**) X-rays until fracture consolidation (**c**) of a fragmentary (OTA 2.B) midshaft clavicle fracture treated using 3.5-mm cortical screw augmentation and locking compression plate

The mean duration of initial surgery was 88.7 ± 29.1 min. The surgical duration was statistically faster (*p* = 0.013) in group I (83.1 ± 19.2) as compared to group II (93.4 ± 35.8). 40 patients received 2.0-mm cortical screw augmentation and 40 patients were treated with 3.5-mm cortical lag screw augmentation. 65 patients were treated using a superior locking compression plate (DePuySynthes, PA, USA) and 15 patients using a superior clavicle fracture plate (Arthrex, Naples, FL, USA). Overall, the mean Fx healing time was 12.8 ± 7.4 months. The time for Fx healing in group I (2.0 mm) accounted for 10.5 ± 5.5 months, whereas a fracture healing time of 14.6 ± 8.5 months was recorded for group II (3.5 mm). Thus, there was no statistically significant difference for fracture healing in group I (2.0 mm) as compared to group II (3.5 mm) (p = 0.10). The type of the used plate osteosynthesis did not influence the time until bony union was achieved (Synthes 13.7 ± 7.7; Arthrex 14.2 ± 3.8; *p* = 0.301). Implant removal was performed upon the patients’ explicit request after a mean of 18.9 ± 7.1 months in 33/80 patients (41.25%, *n* = 31 Synthes, *n* = 2 Arthrex). No mal- or non-unions, as well as no re-Fx of affected clavicles were detected throughout the follow-up appointments, respectively (Fig. 2).

## Discussion

In the present study, we found a high union rate after operative treatment of midshaft clavicle fractures restoring the cortical alignment using 2.0- or 3.5-mm cortical screws, respectively. This rate is most likely allegeable due to increased knowledge with respect to biomechanical properties of the clavicle and the mostly anatomically preformed locking plates.

However, the majority of clavicle Fx can still be treated conservatively, although there has been a clear trend towards operative treatment in the last years [1]. One major complication of conservative treatment in clavicle Fx still is a high rate of non-union (15.1%), which is significantly higher as compared to operative treatment (2.2%) [6, 7]. Furthermore, 15.3% of 122 conservatively treated patients with clavicle fractures were reported with a poor functional outcome after a mean follow-up of 2.7 years [8]. The reported risk factors for delayed fracture consolidation or non-union are smoking [odds ratio (OR) 3.76], degree of dislocation (OR 1.17) and comminuted fractures (OR 1.75) [9, 10]. In contrast to the high OR of smoking (OR 3.76), the degree of dislocation seems to only have menial importance (OR 1.17). From this cohort (941 patients) 125 patients developed non-unions (7.2% (*n* = 52) of 722 nonsmokers/33.3% (*n* = 73) of 219 smokers which goes along with results from Clement et al. [11]. With these numbers Murray et al. presented interesting results since the number needed to treat to prevent a non-union would be 7.5 operative treated clavicle fractures [9, 10]. With respect to comminution several studies have shown less favorable outcomes due to especially high energy trauma [6, 12, 13]. Shortening has been identified as another crucial factor for inferior outcome after conservative treatment with up to 25% unsatisfied patients as described by Lazarides et al. in 2006 and Murray et al., respectively, who both described methods to measure the degree of shortening [4, 10]. Symptomatic non-unions after conservative treatment with lower functional outcomes as well as improved implants may contribute to the significant increase of clavicle fractures of up to 705% over the past two decades, as reported by Huttunen et al. [1]. Also, rising incidence of operative treatment of clavicle Fx was identified for patients > 65 years of age due to a high functional demand in this cohort [14]. Operative treatment can be performed using various fixation devices such as intramedullary fixation or plate osteosynthesis depending on the individual Fx pattern [15–19]. Known indications for surgery include open Fx, displacement of more than 100% or shortening of more than 14 mm in female and 16 mm in male patients or symptomatic non-unions of the Fx [4, 8, 20]. The identification of present Fx patterns is crucial for indicating whether anterior or superior plating should be performed. Biomechanical testing of comminuted Fx revealed superior stability of antero-superior plating compared to anterior plating [19]. In 2018, Hulsman et al. reported on the necessity of restoring anatomic alignment to achieve adequate fixation stability, which is in line with our clinical experience [2]. Restoration of anatomic alignment can sometimes be difficult to achieve in wedge-shaped or multifragmentary clavicle Fx. Fixation using K-wires or other devices can only realize temporary stability. Therefore, in the present study two types of cortical screw augmentation for treating wedge-shaped and fragmentary midshaft clavicle fractures were evaluated. The use of one or more (up to 3) cortical screws was decided depending on the Fx pattern, yet the majority of patients received two cortical screws. From an operative point of view, 2.0-mm screws were easier to handle for the reduction of the fragments compared to 3.5-mm screws, since the greater screw diameter sometimes was cumbersome for smaller fragments. In our study, no mal- or non-unions were recorded, however the mean time until Fx consolidation was 12.8 months. The time until Fx healing appears to be prolonged, but this is owed to our postoperative routine follow-up regimen. Clavicle Fx typically heal within the first 6 months after surgery, yet patients enrolled in this study underwent follow-up visits after 6 and 12 months according to the routine postoperative study protocol [21]. Implant removal was performed after patients’ explicit request after a mean of 18.9 months (*n* = 33). High irritation rates after osteosynthesis of the clavicle are reported in the literature, yet from our experience patients profit from implant removal [5, 22]. Schemetisch et al. reported implant removal to be less often requested when using preformed clavicle plates [23]. However, cortical lag screw augmentation should not be appreciated as a mandatory routine intervention since anatomic alignment can also be achieved using intramedullary or plate fixation without additional lag screw augmentation. However, maintaining reduction of wedge or multifragmentary Fx until plate fixation is often cumbersome and is easier to obtain using (small) cortical screw augmentation. From our point of view, it is a viable method to ease the handling of multifragmentary fractures, which can be helpful for less experienced surgeons. The reported statistically faster operation duration for group I (*p* = 0.013) can be explained by the easier handling of 2.0-mm screws in small fragmentary clavicle fractures, which represents the two senior surgeons’ opinion (P.B, C.K). Obviously, the faster surgical duration is in turn owed to the surgeons’ experience as well as complexity of the fracture itself.

Our study results suggest that cortical lag screw augmentation results in additional fixation stability in wedge-shaped and fragmentary clavicle Fx. In direct comparison, the use of 2.0- and 3.5-mm cortical screws did not show a significant advantage and the diameter of the cortical screw was chosen upon the surgeon’s preference. However, time until Fx healing was shorter in the 2.0-mm group (*p* = 0.10), yet without a statistical significance. Also, the type of the locking compression plate used did not interfere with the time until Fx healing was achieved, yet this study suggests using preformed locking compression plates instead of reconstruction plates, since they showed an inferior outcome from a biomechanical point of view [2, 24].

A limitation of this study is the relatively small patient count and the missing biomechanical testing to elucidate if cortical screw augmentation provides a significant additional stability. These factors need to be addressed by future trials.

## Conclusion

Cortical lag screw augmentation is a viable technique to restore anatomic alignment in displaced wedge-shaped or fragmentary midshaft clavicle Fx. No non-union or re-fracture occurred in both study groups (2.0 vs. 3.5 mm) until fracture healing or after implant removal, which is why complementing an additional cortical lag screw augmentation using 2.0-mm screws can be advised to achieve adequate stability in wedge-shaped and fragmentary midshaft clavicle Fx.

## Data Availability

Further data and material can be accessed by contacting the first/last author.
